# Multiparametric MRI-based radiomics combined with deep learning for preoperative risk stratification of endometrial carcinoma

**DOI:** 10.3389/fonc.2026.1942153

**Published:** 2026-10-22

**Authors:** Yanshan Ouyang, Mianlei Lin, Yiying Cao, Nanxuan He, Xiao Zhong, Yingning Wu

**Affiliations:** 1 Department of Radiology, The First People’s Hospital of Qinzhou, Qinzhou, Guangxi Zhuang Autonomous Region, China; 2 Department of Medical Imaging, Jinjiang Municipal Hospital, Jinjiang, Fujian, China; 3 Department of Radiology, Guigang City People’s Hospital, Guigang, Guangxi Zhuang Autonomous Region, China; 4 Department of Radiology, Affiliated Hospital of Youjiang Medical University for Nationalities, Baise, Guangxi Zhuang Autonomous Region, China

**Keywords:** deep learning, endometrial carcinoma, MRI, radiomics, risk stratification

## Abstract

**Objective:**

To develop and compare radiomics, deep learning, clinical, and combined models based on multiparametric MRI radiomics and 2.5D deep learning features for preoperative risk stratification of endometrial carcinoma (EC).

**Methods:**

This retrospective study included 246 patients with pathologically confirmed EC, with preoperative clinical and imaging data collected. Clinical data, T2-weighted imaging (T2WI), and contrast-enhanced T1-weighted imaging (CE-T1WI) were collected. Radiomics and deep learning models were constructed based on T2WI, CE-T1WI, and their combination. A combined model integrating multiparametric radiomics features, deep learning features, and clinical parameters was subsequently developed. Model performance and clinical utility were evaluated using the area under the receiver operating characteristic curve (AUC), calibration curves, decision curve analysis (DCA), and the DeLong test for pairwise model comparisons. Gradient-weighted Class Activation Mapping (Grad-CAM) was employed to enhance model interpretability.

**Results:**

Univariate and multivariate logistic regression analysis identified maximum tumor diameter, MR-DMI, BMI and ER status as independent predictive factors. Comparison of radiomics, deep learning, clinical, and combined models demonstrated that the multiparametric MRI model outperformed single-sequence models based on radiomics or deep learning, while the combined model exhibited the best predictive performance in the validation set, achieving an AUC of 0.891, accuracy of 0.86, sensitivity of 0.909, specificity of 0.765, and F1-score of 0.896. DeLong test results showed that, in the training set, the combined model had higher predictive performance than all other models, and the differences were statistically significant (P < 0.05). In the validation set, the combined model showed statistically significant improvements compared with the clinical model and the T2+CET1-Rad model (P<0.05). Decision curve analysis indicated that the combined model provided the highest clinical net benefit.

**Conclusion:**

The combination of multiparametric MRI-based radiomics and 2.5D deep learning demonstrated favorable predictive performance in stratifying risk for EC, suggesting its potential utility as a non-invasive tool and offering a promising adjunctive approach to support personalized treatment planning and prognostic evaluation for EC patients in clinical practice.

## 1 Introduction

Endometrial carcinoma (EC) ranks as the sixth most common cancer among women worldwide and is the most prevalent gynecological malignancy in developed countries. Although its incidence significantly exceeds its mortality rate, approximately 97,000 deaths were reported globally in 2020. The risk of EC is closely associated with obesity, metabolic syndrome, and lifestyle factors (1). According to the 2022 European Society for Medical Oncology (ESMO) guidelines, patients were stratified into two risk groups: the low-risk group (corresponding to the “low-risk” category as defined in the guidelines) and the high-risk group (encompassing the “intermediate-risk,” “intermediate-high-risk,” and “high-risk” categories) (2). Low-risk EC includes low-grade endometrioid carcinoma with negative lymphovascular space invasion (LVSI), staged as International Federation of Gynecology and Obstetrics (FIGO) stage IA. High-risk EC is defined as the presence of any of the following: high-grade endometrioid carcinoma, non-endometrioid carcinoma, positive LVSI, positive cervical stromal invasion (CSI), positive lymph node metastasis (LNM), or FIGO stage IB or higher. Accurate risk stratification is crucial for personalized treatment planning and prognostic assessment in patients with EC. Patients with high-risk EC often require more extensive surgical resection and adjuvant radiotherapy or chemotherapy, whereas those with low-risk disease may undergo hysterectomy with bilateral salpingo-oophorectomy without lymphadenectomy, thereby reducing surgical complications such as lymphedema and pelvic neurovascular injury (3–5). Currently, EC risk assessment primarily relies on postoperative histopathological evaluation, including histological type, depth of myometrial invasion, lymphovascular space invasion, and lymph node metastasis status (6). This approach is not only invasive but also limited in capturing the spatial heterogeneity of the tumor. Therefore, there is a clinical need for a non-invasive, whole-tumor sampling technique that can provide timely, comprehensive, and accurate prediction of EC risk stratification.

The integration of big data analytics with medical imaging has given rise to radiomics, an emerging interdisciplinary field. By capturing subtle pathological changes and providing supplementary information on tumor biology and clinical behavior, radiomics enables the construction of refined predictive models and has been widely applied in the non-invasive diagnosis and prognosis prediction of gynecological tumors (7, 8). In recent years, deep learning has been widely adopted in medical image analysis, owing to its superior ability to extract deep latent features from high-dimensional imaging data. Deep learning models have demonstrated significant value in assisting radiologists with disease diagnosis (9, 10), and convolutional neural networks, in particular, have achieved remarkable performance in a variety of image classification tasks. Transfer learning, when applied to deep learning architectures, offers a reliable strategy to address the common challenge of limited sample sizes in medical imaging. This approach enables the construction of high-accuracy predictive models under data-constrained conditions, and has proven effective in enhancing training performance with small datasets (11, 12).

Although both radiomics and deep learning offer distinct advantages, relying on either approach alone may result in incomplete information extraction. The combination of radiomics features and deep learning features holds promise for constructing a more comprehensive predictive model. This study aimed to evaluate the performance of multiparametric MRI-based radiomic models, deep learning models, a clinical model, and a combined model in predicting risk stratification in EC patients, thereby providing a promising adjunctive tool to facilitate individualized treatment decision-making in clinical practice.

## 2 Methods

### 2.1 Study participants

This retrospective study was conducted in accordance with the ethical guidelines of the Declaration of Helsinki and received institutional review board approval, which waived the requirement for written informed consent. Patients with pathologically confirmed EC who underwent surgical treatment between January 2021 and May 2025 were enrolled from two institutions: the Affiliated Hospital of Youjiang Medical University for Nationalities (A) and Baise City People’s Hospital (B). The inclusion criteria were: (1) histopathologically confirmed EC and underwent total hysterectomy; (2) underwent standardized pelvic MRI examination within three weeks prior to surgery. The exclusion criteria were: (1) missing MRI sequences or poor image quality; (2) incomplete clinical or pathological data; (3) receipt of any preoperative therapy (e.g., radiotherapy, chemotherapy, immunotherapy); (4) concurrent diagnosis of other malignancies. Ultimately, 246 patients were included, with 152 from institution A and 94 from institution B. Risk stratification for all patients was uniformly performed according to the 2022 European Society for Medical Oncology (ESMO) guidelines. Based on pathological findings, patients were categorized into low-risk (n=90) and high-risk (n=156) groups, and were randomly divided into a training set (n=196) and a validation set (n=50) at a ratio of 8:2.

Complete clinical and imaging data were collected for all patients, including age, BMI, CA125, CA153, and CA199 levels, tumor size, MR-DMI, MR-CSI, menopausal status, histological type, Ki-67 index, ER, and PR. All clinical data were obtained within three weeks prior to surgery. Histological type, Ki-67 index, ER, and PR were determined from preoperative biopsy specimens. Tumor size was measured as the maximum diameter of the lesion on axial MRI images. All MRI images were independently reviewed by two junior radiologists, and any disagreements were resolved through consensus discussion with a senior radiologist to minimize interpretation bias. All readers were blinded to the final pathological diagnoses to ensure the independence and objectivity of the assessments.

### 2.2 Image acquisition and segmentation of regions of interest

Preoperative pelvic MRI, including both non-contrast and contrast-enhanced sequences, was performed using three scanners: a GE Discovery 750w 3.0T (n=77), a Siemens Amira 1.5T (n=75), and a Siemens 3.0T (n=94). Image acquisition was carried out with matched 16-channel phased-array body coils. The detailed scanning parameters are summarized in **Table 1**.

**Table 1:** MRI acquisition parameters for T2WI and CE-T1WI across three scanners from two centers.

Scanner (institution)	Sequence	TR (ms)	TE (ms)	Field strength (T)	Slice thickness (mm)	FOV (mm)
GE Discovery 750w (A)	T2WI	3798	62	3.0	5.0	300×300
GE Discovery 750w (A)	CE-T1WI	8.4	2.8	3.0	5.0	300×300
Siemens Amira 1.5T (A)	T2WI	6410	95	1.5	5.0	300×300
Siemens Amira 1.5T (A)	CE-T1WI	3.71	1.4	1.5	5.0	300×300
Siemens 3.0T (B)	T2WI	450	12	3.0	5.0	300×300
Siemens 3.0T (B)	CE-T1WI	4.12	2.02	3.0	5.0	300×300

Contrast-enhanced imaging was performed using Gd-DTPA administered at a dose of 0.2 mmol/kg body weight at an injection rate of 2.0–3.0 mL/s.

First, N4 bias field correction was applied to eliminate local grayscale shifts caused by magnetic field inhomogeneity. T2WI and CE-T1WI images were then normalized and resampled to a uniform voxel size of 1mm×1mm×1mm, converting images from different patients to the same resolution and size. Gray-level discretization was performed using a fixed bin width of 100. Then, the preprocessed T2WI and CE-T1WI images were imported into the open-source software ITK-SNAP 3.8.0 (http://www.itksnap.org). A radiologist with over 8 years of experience in pelvic MRI interpretation manually delineated the region of interest (ROI) on each layer along the tumor boundary on axial images. The results were reviewed by another senior radiologist, and any discrepancies were resolved through consensus. The whole flow of the study is shown in **Figure 1**.

**Figure 1:**
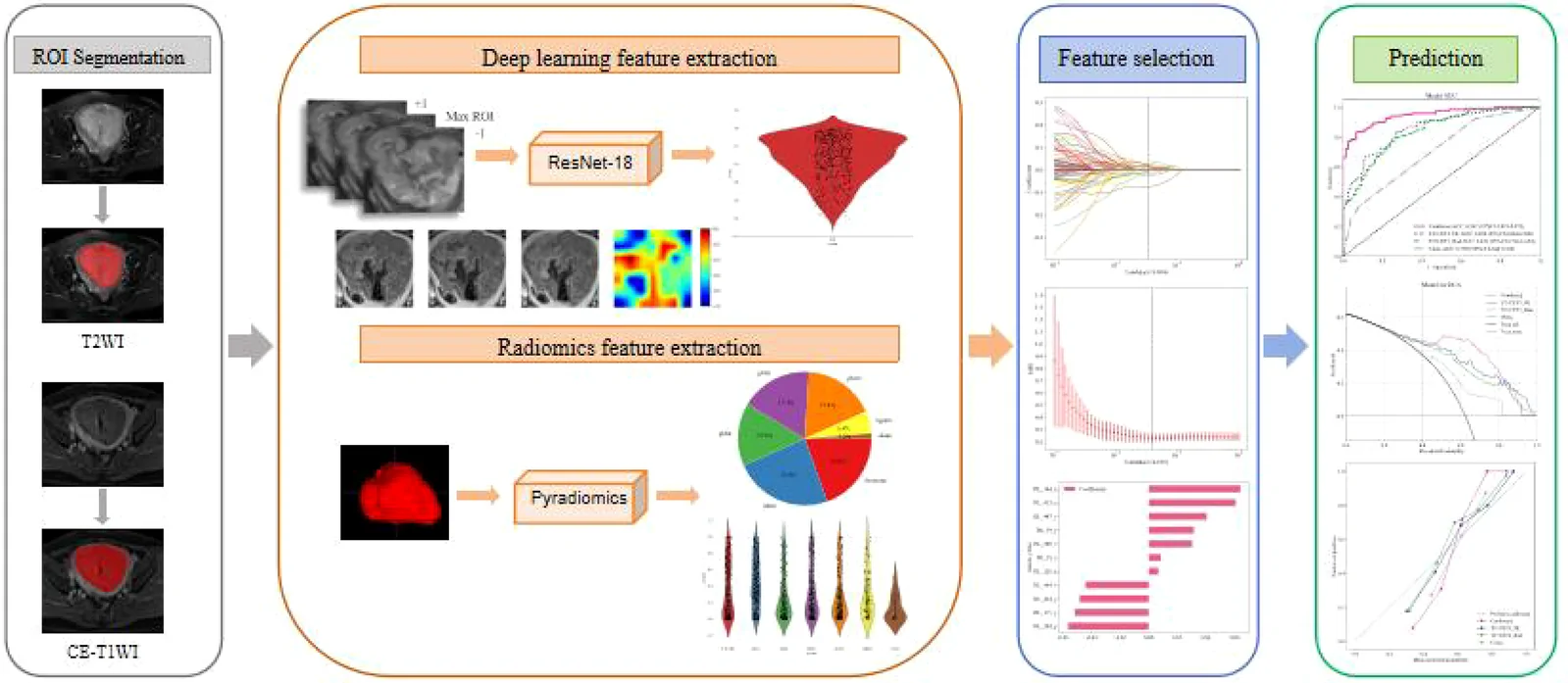
Workflow diagram of the research.

### 2.3 Radiomics features extraction

Radiomic features were extracted using the PyRadiomics open-source software package (version 3.1.0; https://pyradiomics.readthedocs.io/) based on Python 3.7. Multi-dimensional quantitative feature analysis was performed on the delineated ROIs. Features were extracted from 13 image types: the original image (1), wavelet-decomposed images (8: LLL, LLH, LHL, LHH, HLL, HLH, HHL, HHH), and Laplacian of Gaussian (LoG)-filtered images using 3D Gaussian filtering with sigma values ranging from 2.0 to 5.0 mm, totaling 13 image types. A total of 1,197 radiomic features were extracted per sequence, comprising 14 shape features, 65 neighborhood gray-tone difference matrix (NGTDM) features, 234 first-order statistical features, and 884 texture features—including 286 gray-level co-occurrence matrix (GLCM), 208 gray-level run-length matrix (GLRLM), 208 gray-level size zone matrix (GLSZM), and 182 gray-level dependence matrix (GLDM) features.

### 2.4 Deep learning feature extraction

The slice with the largest tumor cross-section and its adjacent upper and lower slices were first cropped as regions of interest. Each cropped subregion was resized to 224×224 pixels using nearest-neighbor interpolation. The three consecutive 2D slices were then stacked along the channel dimension to form a three-channel 2.5D image. A transfer learning approach was adopted using a ResNet-18 architecture pre-trained on the ImageNet database as the backbone network. The pre-trained weights were loaded and transferred to the convolutional neural network in this study to extract deep transfer learning features from the T2WI and CE-T1WI images. Specifically, the 2.5D image data were fed into the network, and deep features were extracted from the penultimate layer (‘avgpool’) as the feature extraction layer, yielding 512 deep learning features from each of the two sequences.

### 2.5 Feature selection

All radiomic and deep learning features extracted from the training set were first standardized using Z-score normalization to convert each feature to a standard normal distribution. Independent samples t-tests were then performed for preliminary feature screening, retaining only those with P < 0.05. Subsequently, Pearson correlation analysis was applied; if the correlation coefficient between two features exceeded 0.9, only one of the two was retained. Finally, the least absolute shrinkage and selection operator (LASSO) algorithm with 10-fold cross-validation was employed for further feature compression, systematically identifying the optimal penalty parameter λ and yielding a final set of features with significant independent predictive value. In the training set, optimal radiomic features (18 for T2WI, 13 for CE-T1WI) and deep learning features (6 for each sequence) were respectively selected to construct single-sequence radiomics and deep learning models. Furthermore, features extracted from the two sequences were fused, and the same feature selection procedure was applied, resulting in 10 and 11 optimal features for the combined radiomics and combined deep learning models (T2WI + CE-T1WI), respectively.

### 2.6 Model construction and validation

Models were constructed using the random forest (RF) machine learning algorithm based on the selected stable and independent radiomic or deep learning features. In the training set, hyperparameter optimization was performed using grid search, and a five-fold cross-validation strategy was employed to prevent overfitting and ensure the selection of optimal parameters with strong generalizability. A clinical model was established using independent clinical risk factors identified through univariate and multivariate regression analysis. A combined model was then constructed by integrating multiparametric radiomic features, 2.5D deep learning features, and clinical features. The validation set was reserved exclusively for final performance evaluation after all model development procedures had been completed. Validation predictions were derived from a single model retrained on the entire training cohort. The predictive performance of each model for EC risk stratification was evaluated using ROC curves, with calculation of the AUC, accuracy, sensitivity, specificity, 95% confidence intervals (95% CI), positive predictive value, negative predictive value, and F1-score. Model calibration was assessed using calibration curves, and clinical utility was evaluated through DCA. The classification threshold was determined using the Youden index, defined as the probability cutoff that maximizes (sensitivity + specificity − 1) in the training set. This threshold was then fixed and applied to the validation set for performance evaluation.

### 2.7 Statistical analysis

All statistical analysis were performed using R (version 4.0.2, https://www.r-project.org) and SPSS 26.0 (IBM, New York, USA). Normally distributed continuous variables were presented as $\bar{x}$ ± S and compared using independent samples t-test. Non-normally distributed continuous variables were expressed as median or interquartile range, and compared with the Mann-Whitney U test. The count data were expressed as the number of cases, and analyzed using the Chi-square test or Fisher’s exact test. All univariate and multivariate analysis were performed using standard binary logistic regression with maximum likelihood estimation (MLE) for coefficient estimation, and the Wald method for calculating odds ratios (ORs), 95% confidence intervals (CIs), and P-values. Differences in the AUC among models were compared using DeLong test. A p-value less than 0.05 was considered statistically significant.

## 3 Results

### 3.1 Clinical data and establishment of clinical model

The clinical and imaging characteristics of EC patients are listed in **Table 2**. In both the training and validation sets, significant differences were observed between the two risk groups in terms of tumor size, MR-DMI, and histological type (P<0.05). Univariate and multivariate regression analysis identified tumor size (OR = 1.041, P = 0.002), MR-DMI (OR = 2.196, P = 0.043), BMI (OR = 0.926, P = 0.043), and ER (OR = 0.149, P = 0.012) as factors significantly associated with risk stratification (P<0.05), as shown in **Table 3**. And these four independent predictive factors were used to construct the clinical model through Logistic regression. The AUC of this model in the training and validation sets were 0.705 and 0.642, respectively, as detailed in **Table 4**. Due to the extreme imbalance in the distribution of histological type between the low-risk and high-risk groups, complete separation occurred for this variable, which prevented logistic regression from producing reliable estimates. Consequently, this variable was not included in the multivariate analysis. PR was not included in the multivariate model due to its high collinearity with ER, as simultaneous inclusion would introduce multicollinearity. Given that ER is positioned more upstream in the hormone signaling pathway, only ER was retained in the multivariate model.

**Table 2:** Clinical imaging data of high-risk and low-risk EC patients in the train set and validation set.

Parameter	Training set (n=196)			Validation set (n=50)		
	Low-risk group	High-risk group	P-value	Low-risk group	High-risk group	P-value
	(n=73)	(n=123)		(n=17)	(n=33)	
Age(year)	53.60 ± 8.56	55.76 ± 5.87	0.016	55.18 ± 8.63	56.39 ± 7.63	0.612
BMI (kg/m²)	25.25 ± 3.65	23.78 ± 3.89	0.009	25.25 ± 5.24	23.85 ± 3.70	0.279
CA125(U/ml)	31.74 ± 30.54	52.46 ± 78.15	0.224	36.03 ± 28.64	106.18 ± 256.76	0.251
CA153(U/ml)	14.70 ± 13.23	17.65 ± 12.98	0.046	16.13 ± 11.69	30.91 ± 54.20	0.164
CA199(U/ml)	47.95 ± 172.24	66.43 ± 162.51	0.012	31.22 ± 63.61	177.89 ± 603.46	0.395
Tumor size(mm)	38.14 ± 11.42	47.33 ± 19.45	<0.001	39.06 ± 15.13	52.03 ± 22.31	0.031
Menopause(n,%)			0.03			0.391
no	34(46.58)	37(30.08)		8(47.06)	10(30.30)	
yes	39(53.43)	86(69.92)		9(52.94)	23(69.70)	
MR-DMI(n,%)			<0.001			0.001
–	58(79.45)	58(47.15)		16(94.12)	14(42.42)	
+	15(20.55)	65(52.85)		1(5.88)	19(57.58)	
MR-CSI(n,%)			0.008			0.62
–	67(91.78)	93(75.61)		16(94.12)	28(84.82)	
+	6(8.22)	30(24.39)		1(5.89)	5(15.15)	
Histological type(n,%)			<0.001			0.005
Endometrioid(G1/2)	73(100.00)	66(53.66)		17(100.00)	19(57.58)	
Endometrioid (G3)	0(0)	45(36.59)		0(0)	14(42.42)	
Non-endometrioid	0(0)	12(9.76)		0(0)	0(0)	
Ki-67(n,%)			0.055			0.017
<50%	49(67.12)	64(52.03)		14(82.35)	14(42.42)	
≥50%	24(32.88)	59(47.97)		3(17.65)	19(57.58)	
ER(n,%)			0.008			1
–	2(2.74)	20(16.26)		2(11.77)	3(9.09)	
+	71(97.26)	103(83.74)		15(88.24)	30(90.91)	
PR(n,%)			0.003			0.225
–	6(8.22)	33(26.83)		1(5.88)	8(24.24)	
+	67(91.78)	90(73.17)		16(94.12)	25(75.76)	

BMI, body mass index; DMI, deep myometrial invasion; CSI, cervical stromal invasion; CA, carbohydrate antigen; ER, estrogen receptor; PR, progesterone receptor.

**Table 3:** Univariate and multivariate regression analysis of clinical data and MRI imaging signs.

Variable	Univariate analysis			Multivariate analysis		
	OR	95%CI	P	OR	95%CI	P
Age	1.010	1.006~1.014	<0.001	1.026	0.990~1.063	0.244
BMI	0.981	0.972~0.991	0.002	0.926	0.869~0.985	0.043
CA125	1.011	1.005~1.016	0.001	1.003	0.996~1.009	0.466
CA153	1.030	1.016~1.044	<0.001	0.998	0.969~1.026	0.892
CA199	1.002	1.000~1.004	0.090			
Tumor size	1.015	1.009~1.020	<0.001	1.041	1.019~1.064	0.002
Menopause	2.026	1.128~3.638	0.018	2.047	0.979~4.276	0.110
MR-DMI	4.333	2.231~8.420	<0.001	2.196	1.160~4.158	0.043
MR-CSI	3.602	1.424~9.110	0.007	2.514	0.894~6.893	0.100
Histological type	1.249e+10	0.000~inf	0.999			
Ki-67	1.882	1.025~3.455	0.041	1.540	0.856~2.770	0.227
ER	0.145	0.033~0.642	0.007	0.149	0.042~0.522	0.012
PR	0.244	0.097~0.615	0.003			

The histological type variable was excluded from the multivariate logistic regression model because it could not be reliably estimated due to complete separation.

**Table 4:** Prediction performance of different models on the training set and validation set.

Models	AUC(95% CI)	Accuracy	Sensitivity	Specificity	PPV	NPV	F1
Training set							
T2_Rad	0.718(0.650 - 0.785)	0.622	0.455	0.904	0.889	0.496	0.602
CET1_Rad	0.748(0.680 - 0.817)	0.689	0.61	0.822	0.852	0.556	0.711
T2+CET1_Rad	0.839(0.784 - 0.894)	0.765	0.77	0.757	0.839	0.667	0.803
T2_DL	0.775(0.710 - 0.840)	0.74	0.837	0.575	0.769	0.677	0.802
CET1_DL	0.781(0.716 - 0.846)	0.694	0.618	0.822	0.854	0.561	0.717
T2+CET1_DL	0.856(0.804 - 0.908)	0.796	0.836	0.73	0.836	0.73	0.836
Clinic	0.705(0.642 - 0.768)	0.556	0.336	0.919	0.872	0.456	0.485
Combined	0.947(0.919 - 0.975)	0.867	0.829	0.932	0.953	0.764	0.887
Validation set							
T2_Rad	0.701(0.555 - 0.847)	0.64	0.545	0.824	0.857	0.483	0.667
CET1_Rad	0.733(0.569 - 0.896)	0.74	0.758	0.706	0.833	0.6	0.794
T2+CET1_Rad	0.764(0.618 - 0.910)	0.72	0.697	0.765	0.852	0.565	0.767
T2_DL	0.714(0.560 - 0.868)	0.74	0.818	0.588	0.794	0.625	0.806
CET1_DL	0.740(0.578 - 0.902)	0.76	0.818	0.647	0.818	0.647	0.818
T2+CET1_DL	0.771(0.627 - 0.915)	0.7	0.606	0.882	0.909	0.536	0.727
Clinic	0.642(0.496 - 0.787)	0.56	0.424	0.824	0.824	0.424	0.56
Combined	0.891(0.800 - 0.982)	0.86	0.909	0.765	0.882	0.812	0.896

Rad, radiomics model; DL, deep learning model; PPV, positive predictive value; NPV, negative predictivevalue.

### 3.2 Construction of radiomics and deep learning models

A total of 18, 13 and 10 optimal radiomics features were selected from T2WI, CE-T1WI, and the combined sequences, respectively. Meanwhile, 6, 6 and 11 optimal deep learning features were selected from the corresponding sequences. Radiomics and deep learning models were constructed using the RF classifier and subsequently validated on the validation set. In the validation set, the AUC values for the radiomics models T2-Rad, CET1-Rad, and T2+CET1-Rad were 0.701, 0.733, and 0.764, respectively. For the deep learning models T2-DL, CET1-DL, and T2+CET1-DL, the AUC values were 0.714, 0.740, and 0.771, respectively (refer to **Table 4**). The results indicated that features extracted from CE-T1WI exhibited superior predictive performance compared to those from T2WI in single-sequence models, while the combined-sequence model demonstrated superior performance compared to any single-sequence model. Furthermore, the deep learning models achieved higher AUC values than the radiomics models in both the training and validation sets. ROC curves for the different models are presented in **Figure 2**.

**Figure 2:**
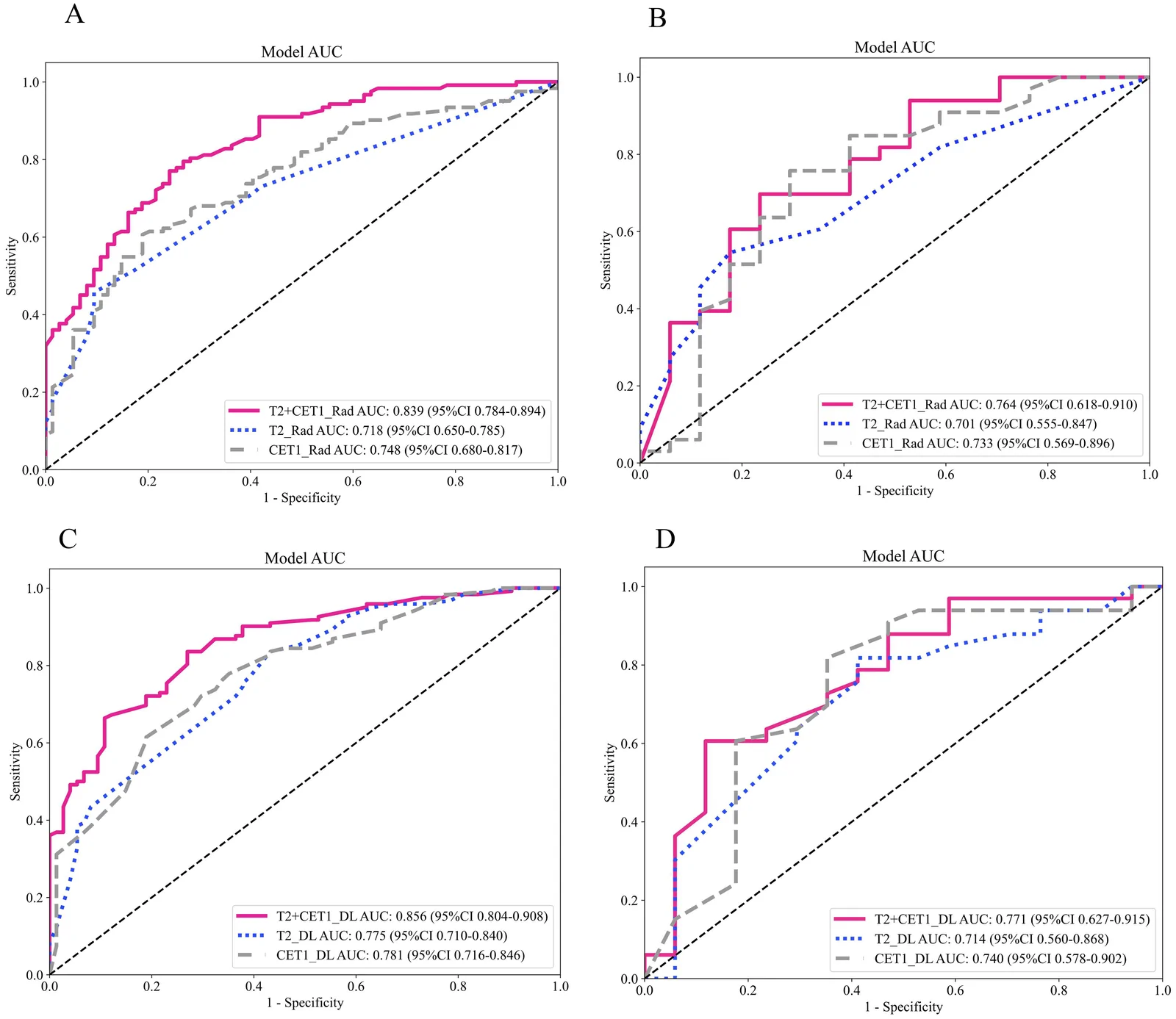
ROC curves of single-sequence and multi-sequence fusion imaging biomarker models for the training set **(A)** and validation set **(B)**; the T2+CET1-Rad model demonstrated higher AUC values than the single-sequence radiomics models. ROC curves of single-sequence and multi-sequence fusion deep learning models for the training set **(C)** and validation set **(D)**; the T2+CET1-DL model demonstrated higher AUC values than the single-sequence deep learning models.

Based on the feature maps of the final convolutional layer, the corresponding weights for each channel were derived using the backpropagation mechanism, and the resulting weights were subsequently multiplied by the feature maps to generate gradient-weighted class activation mapping (Grad-CAM) for visualization purposes, thereby enhancing the transparency of the model’s decision-making mechanism and intuitively highlighting the critical image regions contributing most to the classification outcomes, as shown in **Figure 3**.

**Figure 3:**
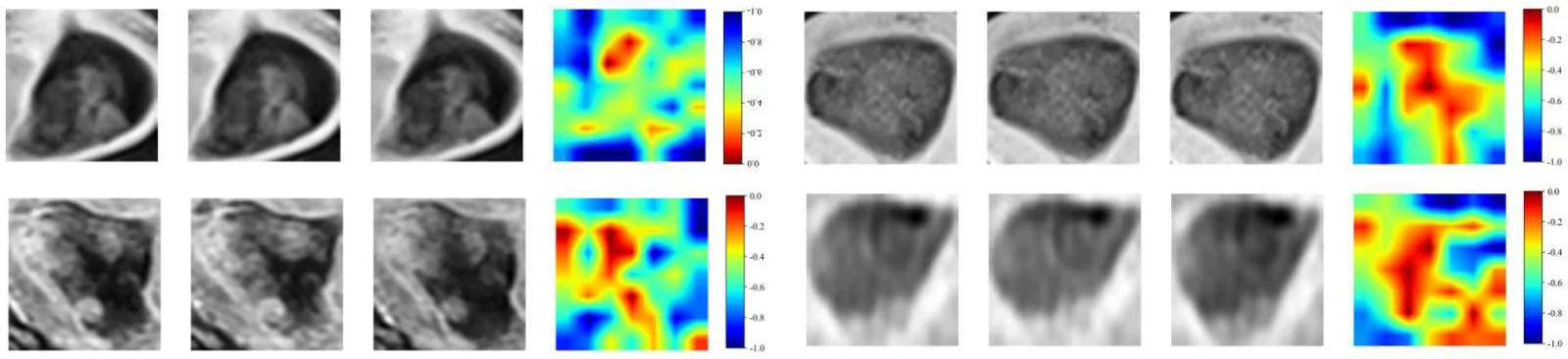
Representative Grad-CAM visualizations of the T2-DL model for correctly classified cases from the validation set. The upper two panels show low-risk cases, and the lower two panels show high-risk cases. In the heatmaps, red areas indicate the highest contribution to the model’s decision, representing the regions most critical for classification; dark blue areas indicate the lowest contribution.

### 3.3 Combined model construction and model performance

Radiomics features, deep learning features, and clinical features were concatenated to form a comprehensive feature set. Feature selection and dimensionality reduction were performed using the methods described above, and a combined model was constructed using the RF classifier. The results showed that the combined model achieved the best in risk prediction, with its AUC values (0.947, 0.891), accuracy (0.867, 0.860), sensitivity (0.829, 0.909), and F1 values (0.887, 0.896) all showing better overall classification performance numerically in both the training and validation sets (**Table 4**). The F1 value was superior to those of the other comparative models, indicating better performance in positive sample identification and classification error control. The ROC curves, DCA, DeLong test results, and calibration curves for the combined model, the T2+CET1-DL model, the T2+CET1-Rad model, and the clinical model are presented in **Figure 4**. DeLong test showed that in the training set, the predictive efficacy of the combined model was significantly superior to that of the radiomics, deep learning, and clinical models. In the validation set, there were statistically significant differences between the combined model and the clinical model, as well as between the combined model and the T2+CET1-Rad model (P<0.05) (**Figures 4C, D**). DCA indicated that the combined model provided higher net benefit and better clinical decision-making utility compared to the clinical, radiomics, and deep learning models (**Figures 4E, F**). Calibration curve analysis demonstrated that the combined model’s calibration curve most closely approximated the ideal diagonal, indicating good agreement between predicted probabilities and observed outcomes. This suggests acceptable calibration performance and supports the model’s potential utility as a clinical decision-support tool (**Figures 4G, H**).

**Figure 4:**
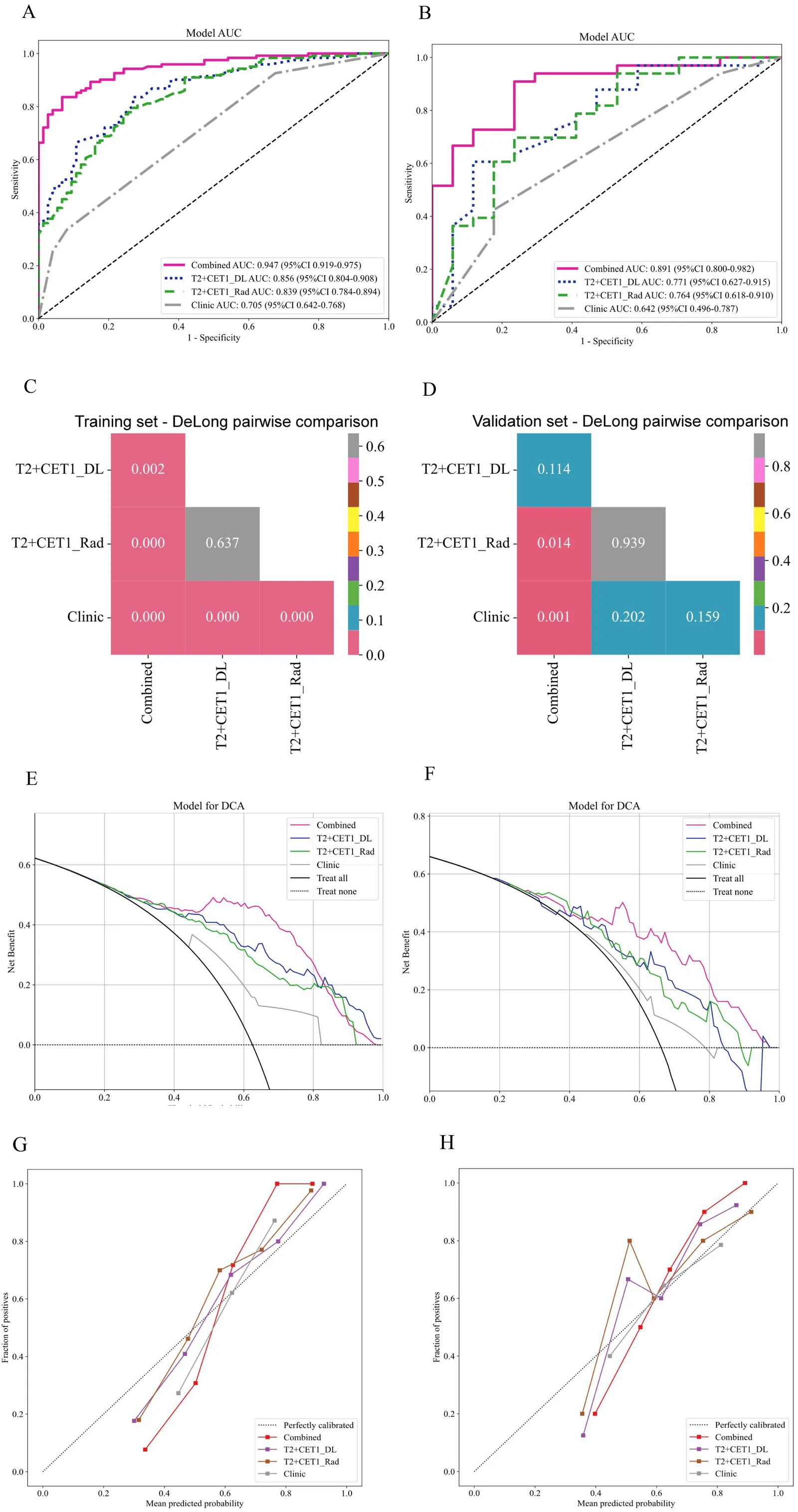
ROC curves of the four models in the training set **(A)** and validation set **(B)**. DeLong test of the four models in the training set **(C)** and validation set **(D)**. DCA diagrams of the four models in the training set **(E)** and validation set **(F)**. Calibration curves of the four models in the training set **(G)** and validation set **(H)**.

## 4 Discussion

MRI provides high soft-tissue resolution and multi-parametric imaging capabilities, it can clearly display the size, location and relationship of EC with surrounding tissues (13, 14). In recent years, deep learning has demonstrated considerable advantages in medical research, overcoming the limitations of traditional qualitative or semi-quantitative imaging analysis through end-to-end feature learning, thereby offering new opportunities for MRI-based EC diagnosis (15–17). In this study, radiomics and deep learning features were extracted from T2WI and CE-T1WI of EC patients. Combined with conventional MRI images and clinical data, eight models were developed for preoperative risk stratification, including clinical model, single-sequence and multi-sequence radiomics and deep learning models, and integrated feature models. The combined model achieved the highest AUC values in both the training (AUC = 0.947) and validation (AUC = 0.891) sets. DeLong test results showed that, in the training set, the combined model significantly outperformed all other models (P<0.05). In the validation set, the combined model demonstrated statistically significant superiority over some, but not all, comparators (P<0.05), with no significant difference observed against the T2+CET1-DL model (P = 0.114). These findings suggest that the combined model holds promise for preoperative risk stratification in endometrial carcinoma; however, its generalizability and superiority require further confirmation in larger, external validation cohorts.

The enrollment period of this study spanned from 2021 to 2025, during which the 2022 ESMO guidelines were updated. To ensure consistency in risk classification throughout the study period, all cases were retrospectively reviewed, and complete clinicopathological data were collected and uniformly re-evaluated according to the 2022 ESMO risk stratification criteria. However, patient management strategies—such as surgical approaches and adjuvant therapy recommendations—may have evolved over time in clinical practice. Although these potential temporal variations may have some influence on treatment decisions and prognostic assessment, their impact on the predictive performance of our model is likely limited. Future studies with prospective multi-center cohorts are warranted to further validate the stability and generalizability of our model under unified management protocols.

An unexpected finding of this study was that low BMI emerged as an independent risk factor for high-risk endometrial carcinoma (OR = 0.926, P = 0.043), which appears to contrast with the established consensus that obesity is a major risk factor for this malignancy. This discrepancy may be attributable to heterogeneity in the study population. Notably, our high-risk group contained a relatively large proportion of patients with advanced-stage disease, who typically present with greater tumor burden and deeper myometrial invasion. In this context, low BMI may reflect tumor-related cachexia—a systemic metabolic state commonly associated with advanced malignancies, characterized by involuntary weight loss, muscle wasting, and malnutrition. Thus, low BMI in our study may serve as a marker of disease severity rather than a conventional etiological factor. Nevertheless, this finding requires further validation through multicenter, large-scale, prospective cohort studies.

Previous studies have demonstrated the advantages of multi-phase enhanced scanning, indicating its higher specificity compared to T2WI in evaluating myometrial invasion and cervical stromal involvement (18). Our study also incorporated CE-T1WI sequences, and the results demonstrated superior predictive performance for EC risk stratification compared to the T2WI model. The majority of research has shown that multi-sequence fusion models or integrated models outperform single-sequence models in predicting pelvic lymph node metastasis(LNM), lymphovascular space invasion(LVSI), and risk stratification of EC (19–21). Lefebvre et al. (22) developed a random forest (RF) model based on multiparametric MRI for preoperative EC risk stratification, achieving an AUC ranging from 0.74 to 0.81. Yang et al. (23) combined conventional radiomics features, deep learning features, and clinical parameters from multiparametric MRI to construct a preoperative risk classification model, which yielded an AUC of 0.923 in the training set and 0.842 in the validation set. Compared with their study, our work has several distinguishing features. First, our study enrolled 246 patients, providing a larger sample size and thus an advantage in assessing model robustness, with a validation AUC of 0.891. Second, we incorporated CE-T1WI sequences, which reflect tumor vascularity and perfusion, potentially offering complementary imaging information for risk stratification. Third, we included a broader range of clinical variables—such as BMI, CA153, CA199, menopausal status, ER, and PR—enabling a more comprehensive clinical-imaging feature profile, which may contribute to improved predictive performance. Fourth, we adopted a ResNet-18 architecture, which balances model expressiveness with computational efficiency.

The above research results all indicate that the combined model has the best prediction performance, which is consistent with our findings. The combined model constructed in this study exhibited significant advantage, with an AUC of 0.891 (95% CI: 0.800–0.982) and a sensitivity of 0.909 in the validation set. The DeLong test demonstrated that in the training set, the predictive performance of the combined model was significantly superior to that of the radiomics, deep learning, and clinical models. This indicates that the combined model effectively synthesizes multi-source information, enabling a multi-dimensional assessment of patient status. It can more comprehensively reflect the biological characteristics of the lesion, thereby improving the prediction accuracy.

In recent years, convolutional neural networks have demonstrated outstanding capabilities in feature extraction and structural representation within medical image analysis. ResNet-18, as the main architecture of the transfer learning model, its lightweight structure and stable performance have been verified in multiple studies (24, 25). Compared with deeper networks, ResNet-18 maintains a high level of feature representation capability while reducing computational complexity and the risk of overfitting. In this study, ResNet-18 was employed as the core framework for multi-sequence deep feature extraction to develop a predictive model. The T2+CET1-DL model, which integrated features from both sequences, achieved AUC values of 0.856 and 0.771 in the training and validation sets, respectively, demonstrating its ability to effectively capture radiomic representations associated with risk stratification from multiple dimensions. Multiple studies have indicated that deep transfer learning methods perform well in predicting key pathological indicators of EC, such as CSI and DMI (26–28). Qi et al. (29) developed a residual network (ResNet-101) based on the deep learning convolutional neural network (CNN) for predicting high-risk EC, achieving an AUC of 0.918, which significantly outperformed traditional ResNet-101 (AUC = 0.613), SA-ResNet-101 (AUC = 0.760), and CA-ResNet-101 (AUC = 0.758) models, and also effectively predicted postoperative recurrence.

The “black box” nature of deep learning models has always been a key factor restricting their clinical wide application. To further enhance model interpretability, this study employed the Grad-CAM technique to generate visual heatmaps, which visually demonstrated the model’s attention to tumor regions (such as the lesion margins and internal structures), thereby establishing a visual correlation between imaging features and risk levels (30). The heat map results of this study show that the deep learning model pays particular attention to the internal signal heterogeneity regions of the tumor when making risk classification judgments. These regions are often closely related to the invasive biological behavior of the tumor and enhance clinical interpretability (31). These results not only demonstrate the potential of deep learning models in imaging informatics, but also provide important reference evidence for clinical practice. Future research can further explore multi-scale feature fusion strategies combined with attention mechanisms to enhance the model’s sensitivity to key pathological features.

This study also has several limitations. First, MRI data were acquired using scanners from different vendors and field strengths (1.5T and 3.0T). Although N4 bias field correction was applied during preprocessing to reduce intra-image intensity inhomogeneity, this method is primarily designed to address multiplicative artifacts within a single image and cannot fully eliminate inherent signal intensity differences across field strengths. Such scanner-related heterogeneity may have a potential impact on radiomic feature reproducibility. Therefore, the influence of scanner heterogeneity on model performance should be considered when interpreting our results. Future studies may incorporate batch-effect harmonization methods such as ComBat or validate model robustness in larger multi-center cohorts. Second, although this study included cases from two institutions, external validation was not performed. Further work with expanded sample sizes and multi-regional cohorts is warranted to strengthen the generalizability of our findings. Third, deep learning features were extracted from three representative slices rather than the entire tumor volume. While this approach offers advantages in clinical practicality and computational efficiency, it may not fully capture the spatial heterogeneity of the tumor. Fourth, inter-reader kappa statistics for MR-DMI and MR-CSI were not formally assessed. Although independent review and consensus were performed, this process does not replace formal reliability testing. The absence of kappa data may affect reproducibility and should be considered when interpreting our findings. Future work should include formal kappa analysis. Only T2WI and CE-T1WI sequences were included. Although these sequences are widely used in clinical practice, adding DWI, ADC scanning parameters, or multi-modal research in the future can further uncover more valuable features.

## 5 Conclusion

In conclusion, the combined model integrating multiparametric radiomic, deep learning, and clinical features demonstrated favorable predictive performance in stratifying risk for EC, suggesting its potential utility as a non-invasive tool and offering a promising adjunctive approach to support personalized treatment planning and prognostic evaluation for EC patients in clinical practice.

## Data Availability

The original contributions presented in the study are included in the article/supplementary material. Further inquiries can be directed to the corresponding author.

## References

[1] Sung H Ferlay J Siegel RL Laversanne M Soerjomataram I Jemal A . Global cancer statistics 2020: GLOBOCAN estimates of incidence and mortality worldwide for 36 cancers in 185 countries. CA: A Cancer J For Clin. (2021) 71:209–49. doi: 10.3322/caac.21660 33538338

[2] Oaknin A Bosse TJ Creutzberg CL Giornelli G Harter P Joly F . Endometrial cancer: ESMO clinical practice guideline for diagnosis, treatment and follow-up. Ann Oncol Off J Eur Soc For Med Oncol. (2022) 33:860–77. doi: 10.1016/j.annonc.2022.05.009 35690222

[3] Brooks RA Fleming GF Lastra RR Lee NK Moroney JW Son CH . Current recommendations and recent progress in endometrial cancer. CA: A Cancer J For Clin. (2019) 69:258–79. doi: 10.3322/caac.21561 31074865

[4] Concin N Matias-Guiu X Vergote I Cibula D Mirza MR Marnitz S . ESGO/ESTRO/ESP guidelines for the management of patients with endometrial carcinoma. Int J Gynecological Cancer: Off J Int Gynecological Cancer Soc. (2021) 31:12–39. doi: 10.1016/j.radonc.2020.11.018 33397713

[5] Gorzelnik K Szubert S Knafel A Wójcikiewicz A Nowakowski B Koper K . An analysis of the significance of the lymph node ratio and extracapsular involvement in the prognosis of endometrial cancer patients. Contemp Oncol (Poznan Poland). (2022) 26:144–9. doi: 10.5114/wo.2022.118243 35903209 PMC9319184

[6] Imboden S Nastic D Ghaderi M Rydberg F Siegenthaler F Mueller MD . Implementation of the 2021 molecular ESGO/ESTRO/ESP risk groups in endometrial cancer. Gynecologic Oncol. (2021) 162:394–400. doi: 10.1016/j.ygyno.2021.05.026 34127276

[7] Aerts HJ Velazquez ER Leijenaar RT Parmar C Grossmann P Carvalho S . Decoding tumour phenotype by noninvasive imaging using a quantitative radiomics approach. Nat Commun. (2014) 5:4006. doi: 10.1038/ncomms5006 24892406 PMC4059926

[8] Perelli F Mattei A Scambia G Cavaliere AF Editorial: Methods in gynecological oncology. Front Oncol. (2023) 13:1167088. doi: 10.3389/fonc.2023.1167088 36969075 PMC10036035

[9] Gu B Meng M Xu M Feng DD Bi L Kim J . Multi-task deep learning-based radiomic nomogram for prognostic prediction in locoregionally advanced nasopharyngeal carcinoma. Eur J Nucl Med Mol Imaging. (2023) 50:3996–4009. doi: 10.1007/s00259-023-06399-7 37596343 PMC10611876

[10] Theodosiou AA Read RC . Artificial intelligence, machine learning and deep learning: Potential resources for the infection clinician. J Infection. (2023) 87:287–94. doi: 10.1016/j.jinf.2023.07.006 37468046

[11] Guo W Dong Y Hao G . Transfer learning empowers accurate pharmacokinetics prediction of small samples. Drug Discov Today. (2024) 29:103946. doi: 10.1016/j.drudis.2024.103946 38460571

[12] Toseef M Olayemi Petinrin O Wang F Rahaman S Liu Z Li X . Deep transfer learning for clinical decision-making based on high-throughput data: Comprehensive survey with benchmark results. Briefings Bioinf. (2023) 24:bbad254. doi: 10.1093/bib/bbad254 37455245

[13] Fasmer KE Bjørnerud A Ytre-Hauge S Grüner R Tangen IL Werner HM . Preoperative quantitative dynamic contrast-enhanced MRI and diffusion-weighted imaging predict aggressive disease in endometrial cancer. Acta Radiologica (Stockholm Sweden 1987). (2018) 59:1010–7. doi: 10.1177/0284185117740932 29137496

[14] Kido A Himoto Y Kurata Y Minamiguchi S Nakamoto Y . Preoperative imaging evaluation of endometrial cancer in FIGO 2023. J Magnetic Resonance Imaging: JMRI. (2024) 60:1225–42. doi: 10.1002/jmri.29161 38146775

[15] Bhardwaj V Sharma A Parambath SV Gul I Zhang X Lobie PE . Machine learning for endometrial cancer prediction and prognostication. Front Oncol. (2022) 12:852746. doi: 10.3389/fonc.2022.852746 35965548 PMC9365068

[16] Heo T Lee NK Kim S Hong SB Suh DS Kim JY . Deep learning reconstruction of diffusion-weighted imaging with single-shot echo-planar imaging in endometrial cancer: A comparison with multi-shot echo-planar imaging. Abdominal Radiol (New York). (2025) 50:5446–56. doi: 10.1007/s00261-025-04955-3 40249551

[17] Wang Z Hu Y Cai J Xie J Li C Wu X . Potential value of novel multiparametric MRI radiomics for preoperative prediction of microsatellite instability and Ki-67 expression in endometrial cancer. Sci Rep. (2025) 15:3226. doi: 10.1038/s41598-025-87966-w 39863695 PMC11762281

[18] Maheshwari E Nougaret S Stein EB Rauch GM Hwang KP Stafford RJ . Update on MRI in evaluation and treatment of endometrial cancer. Radiographics: A Rev Publ Radiological Soc North America Inc. (2022) 42:2112–30. doi: 10.1148/rg.220070 36018785

[19] Luo Y Mei D Gong J Zuo M Guo X . Multiparametric MRI-based radiomics nomogram for predicting lymphovascular space invasion in endometrial carcinoma. J Magnetic Resonance Imaging: JMRI. (2020) 52:1257–62. doi: 10.1002/jmri.27142 32315482

[20] Yan BC Li Y Ma FH Feng F Sun MH Lin GW . Preoperative assessment for high-risk endometrial cancer by developing an MRI- and clinical-based radiomics nomogram: A multicenter study. J Magnetic Resonance Imaging: JMRI. (2020) 52:1872–82. doi: 10.1002/jmri.27289 32681608

[21] Yan BC Li Y Ma FH Zhang GF Feng F Sun MH . Radiologists with MRI-based radiomics aids to predict the pelvic lymph node metastasis in endometrial cancer: A multicenter study. Eur Radio. (2021) 31:411–22. doi: 10.1007/s00330-020-07099-8 32749583

[22] Lefebvre TL Ueno Y Dohan A Chatterjee A Vallières M Winter-Reinhold E . Development and validation of multiparametric MRI-based radiomics models for preoperative risk stratification of endometrial cancer. Radiology. (2022) 305:375–86. doi: 10.1148/radiol.212873 35819326

[23] Yang J Cao Y Zhou F Li C Lv J Li P . Combined deep-learning MRI-based radiomic models for preoperative risk classification of endometrial endometrioid adenocarcinoma. Front Oncol. (2023) 13:1231497. doi: 10.3389/fonc.2023.1231497 37909025 PMC10613647

[24] Liu X Zhang D Liu Z Li Z Xie P Sun K . Deep learning radiomics-based prediction of distant metastasis in patients with locally advanced rectal cancer after neoadjuvant chemoradiotherapy: A multicentre study. EBioMedicine. (2021) 69:103442. doi: 10.1016/j.ebiom.2021.103442 34157487 PMC8237293

[25] Wang F Chen Q Chen Y Zhu Y Zhang Y Cao D . A novel multimodal deep learning model for preoperative prediction of microvascular invasion and outcome in hepatocellular carcinoma. Eur J Surg Oncol J Eur Soc Surg Oncol Br Assoc Surg Oncol. (2023) 49:156–64. doi: 10.1016/j.ejso.2022.08.036 36333180

[26] Wang X Bi Q Deng C Wang Y Miao Y Kong R . Multiparametric MRI-based radiomics combined with 3D deep transfer learning to predict cervical stromal invasion in patients with endometrial carcinoma. Abdominal Radiol (New York). (2025) 50:1414–25. doi: 10.1007/s00261-024-04577-1 39276192

[27] Xiong L Chen C Lin Y Mao W Song Z . A computer-aided determining method for the myometrial infiltration depth of early endometrial cancer on MRI images. BioMed Eng Online. (2023) 22:103. doi: 10.1186/s12938-023-01169-w 37907955 PMC10617104

[28] Yue W Han R Wang H Liang X Zhang H Li H . Development and validation of clinical-radiomics deep learning model based on MRI for endometrial cancer molecular subtypes classification. Insights into Imaging. (2025) 16:107. doi: 10.1186/s13244-025-01966-y 40377781 PMC12084453

[29] Qi X . Artificial intelligence-assisted magnetic resonance imaging technology in the differential diagnosis and prognosis prediction of endometrial cancer. Sci Rep. (2024) 14:26878. doi: 10.1038/s41598-024-78081-3 39506051 PMC11541869

[30] M MM T R M V VK Guluwadi S . Enhancing brain tumor detection in MRI images through explainable AI using Grad-CAM with Resnet 50. BMC Med Imaging. (2024) 24:107. doi: 10.1186/s12880-024-01292-7 PMC11088067 38734629

[31] Wang S Ren J Guo X . A high-accuracy lightweight network model for X-ray image diagnosis: A case study of COVID detection. PloS One. (2024) 19:e0303049. doi: 10.1371/journal.pone.0303049 38889106 PMC11185471

